# The Known Antimammalian and Insecticidal Alkaloids Are Not Responsible for the Antifungal Activity of *Epichloë* Endophytes

**DOI:** 10.3390/plants10112486

**Published:** 2021-11-17

**Authors:** Krishni Fernando, Priyanka Reddy, Simone Vassiliadis, German C. Spangenberg, Simone J. Rochfort, Kathryn M. Guthridge

**Affiliations:** 1 Agriculture Victoria, AgriBio, Centre for AgriBioscience, Bundoora, VIC 3083, Australia; krishni.fernando@agriculture.vic.gov.au (K.F.); priyanka.reddy@agriculture.vic.gov.au (P.R.); simone.vassiliadis@agriculture.vic.gov.au (S.V.); german.spangenberg@agriculture.vic.gov.au (G.C.S.); simone.rochfort@agriculture.vic.gov.au (S.J.R.); 2 School of Applied Systems Biology, La Trobe University, Bundoora, VIC 3083, Australia

**Keywords:** *Epichloë* sp. endophytes, pasture protection, turf, disease control, alkaloids, antifungal activity, bioprotection

## Abstract

Asexual *Epichloë* sp. endophytes in association with pasture grasses produce agronomically important alkaloids (e.g., lolitrem B, epoxy-janthitrems, ergovaline, peramine, and lolines) that exhibit toxicity to grazing mammals and/or insect pests. Novel strains are primarily characterised for the presence of these compounds to ensure they are beneficial in an agronomical setting. Previous work identified endophyte strains that exhibit enhanced antifungal activity, which have the potential to improve pasture and turf quality as well as animal welfare through phytopathogen disease control. The contribution of endophyte-derived alkaloids to improving pasture and turf grass disease resistance has not been closely examined. To assess antifungal bioactivity, nine *Epichloë* related compounds, namely peramine hemisulfate, *n*-formylloline-d3, *n*-acetylloline hydrochloride, lolitrem B, janthitrem A, paxilline, terpendole E, terpendole C, and ergovaline, and four *Claviceps purpurea* ergot alkaloids, namely ergotamine, ergocornine, ergocryptine, and ergotaminine, were tested at concentrations higher than observed in planta in glasshouse and field settings using in vitro agar well diffusion assays against three common pasture and turf phytopathogens, namely *Ceratobasidium* sp., *Drechslera* sp., and *Fusarium* sp. Visual characterisation of bioactivity using pathogen growth area, mycelial density, and direction of growth indicated no inhibition of pathogen growth. This was confirmed by statistical analysis. The compounds responsible for antifungal bioactivity of *Epichloë* endophytes hence remain unknown and require further investigation.

## 1. Introduction

Asexual *Epichloë* sp. are endophytic fungi that play a critical role in pastoral agricultural systems as they enhance pasture performance. Improved growth in pastures is achieved in part from the bioprotective properties endophytes confer, including pest and disease resistance as well as abiotic stress tolerance [1,2,3,4]. While the impact of alkaloids on animal performance and insect control is well understood [5,6,7,8], the bioprotective compounds responsible for improved pasture quality and performance, in particular disease control, have not been thoroughly investigated.

Pasture and turf grasses are threatened by many phytopathogens, including fungi, bacteria, and viruses. Severe disease outbreaks impact pasture quality and yield [9,10,11]. Adding to the effect on the dairy and meat industries by reduced yields, mycotoxins produced by some phytopathogenic fungi are a health threat to grazing mammals [12,13,14]. Better understanding of *Epichloë*-mediated disease resistance, and identifying the compounds responsible, provides an opportunity to identify *Epichloë* endophyte strains better suited for pastoral agriculture and turf systems. 

*Epichloë* sp. are able to improve disease resistance of host grasses by reducing pathogen incidence and/or disease severity [3,15,16,17]. Historically, the potential to improve disease resistance in pastures using endophytes has been investigated in vitro. This was achieved by assessing antifungal activity of different strains of *Epichloë* sp. against phytopathogens in dual culture, detached leaf, and spore germination assays [1,3,18,19,20]. The in vitro assays confirm the activity against a range of phytopathogens, e.g., *Alternaria alternata*, *Bipolaris sorokiniana, Colletotrichum graminicola, Drechslera erythrospila, Ceratotobasidium cereale (Rhizoctonia cerealis), Rhizoctonia zeae,* and *Fusarium* sp. [3,20,21]. There are also examples where endophyte control of biotrophic fungi, such as *Puccinia coronata* (crown rust), are evaluated [19,22]. Other studies evaluated in vitro culture filtrates and culture extracts of *Epichloë* sp. endophyte strains [20,21]. In these studies, the potential for antifungal compound production by *Epichloë* sp. endophytes was assessed with use of agar well diffusion assays and thin layer chromatography (TLC) bioautography assays [20,21,23]. 

While the asexual (non-pathogenic) *Epichloë* sp. utilised in pasture and turf grasses have not been investigated with the same level of detail, bioactive antifungal compounds have been identified and isolated from sexual (pathogenic) *Epichloë* sp. Antifungal compounds that have been identified and isolated from *Epichloë typhina* include chokols (A–G), gamahonolide A, gamahonolide B, gamahorin, and epichlicin [24,25,26,27]. Several studies also describe isolation of antifungal agents from *Epichloë festucae*. An antifungal protein (55 amino acids, 6.3 kDa) named Efe-AfpA was isolated from *E. festucae* (Rose City isolate) and was shown to inhibit the growth of *Sclerotinia homoeocarpa* in a plate assay. Another antibiotic protein, VibA, was identified when the gene was overexpressed in *E. festucae* strain E437. The authors determined the *VibA* gene encoded for a biosynthetic enzyme that produced ε-poly-l-lysines of 28–34 lysine subunits. The antibiotic metabolites indole-3-acetic acid, indole-3-ethanol, methylindole-3-carboxylate, indole-3-carboxaldehyde, *N,N*-diacetamide, cyclonerodiol (an isomer of chokol C) have been isolated from *E. festucae* (BM7, M. D. Richardson) and showed inhibition against *Cryphonectria parasitica*, *Magnaporthe poae*, *Laetisaria fuciformis,* and *Rhizoctonia solani* [17,21,28,29].

Fernando et al. (2020) previously identified variation for antifungal activity between different strains of asexual *Epichloë* sp. isolated from perennial ryegrass (*Lolium perenne*) and tall fescue (*Festuca arundinacea*) plants [20,30,31]. The study showed that unique compounds produced by three highly bioactive endophyte strains, namely SE, NEA12, and NEA23, may be associated with the distinct antifungal activity exhibited by each strain. In addition to the still-to-be isolated and characterised unique compounds, these strains have been assessed for in planta production of alkaloids including lolitrem B, ergovaline, peramine, *n*-acetylloline, *n*-formylloline and epoxy janthitrems. These alkaloids are usually only considered in their role as mammalian or insect toxins. However, there is a possibility that these alkaloids contribute to the observed antifungal activity. 

Only one study, in 1991, has tested *Epichloë* sp. derived alkaloids for their antifungal potential. Seigel and Latch used disk diffusion assays to evaluate *n*-formylloline, *n*-acetylloline, peramine sulfate, ergonovine maleate, ergotamine tartrate, and ergocryptine (100 mg/disk) for bioactivity against the phytopathogens *Ceratobasidium cereale, Colletotrichum graminicola, Rhizoctonia zeae,* and *Limonomyces roseipellis,* and in each instance they did not observe bioactivity despite the high concentrations of each compound used in the assay [18]. 

While the study revealed no significant antifungal activity from these compounds it does not account for three major alkaloids, ergovaline, lolitrem B and epoxy-janthitrem I or indole diterpene biosynthetic intermediates paxilline, terpendole E, and terpendole C. In the last decade, epoxy-janthitrems, a class of indole diterpenes that are structurally related to lolitrem B, were identified in *Epichloë* sp. endophyte strains including NEA12. Due to the inherent instability of pure epoxy janthitrems, stable isolation and purification remain elusive [32]. Consequently, there are limited reports on their sound biological activity in insects and mammals [33]. Since the study in 1991, other relevant purified compounds have also become available, thus providing an opportunity to screen and quantify a wider range of compounds for their bioprotective properties.

This study analyses the antifungal activity of thirteen *Epichloë* sp. related compounds against the phytopathogens *Ceratobasidium* sp., *Fusarium* sp., and *Drechslera* sp. to determine their contribution to the observed bioactivity previously described in *Epichloë* sp. endophyte strains by Fernando et al. (2020) [20].

## 2. Results

Bioprotective alkaloids known to be produced by *Epichloë* sp. strains utilised in pastures and turf were obtained from either commercial or in-house sources to assess their antifungal activity toward three phytopathogens *Ceratobasidium* sp., *Drechslera* sp., and *Fusarium* sp. (Table 1) in an agar well diffusion assay. Concentrations well above those observed in planta were prepared for each compound to provide the best prospects of detecting antifungal activity (Table 2). The agar well diffusion assay results provide a visual representation of biological activity exhibited by these alkaloids. Antifungal activity is indicated by changes in the pathogen growth area, mycelial density, and direction of growth [20]. Image analysis using ImageJ software enables quantification of the activity by accurately measuring the pathogen growth area. The growth area measurements can then be used for statistical analysis.

**Figure 1:**
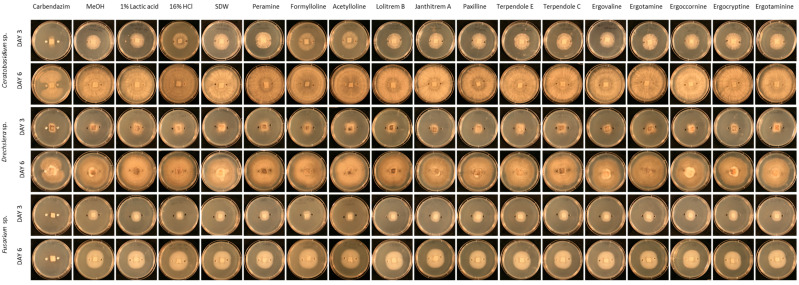
Optimised agar-well diffusion assay for growth inhibition of pathogens *Ceratobasidium* sp., *Drechslera* sp. and *Fusarium* sp. by thirteen alkaloids and controls (in columns); carbendazim, 80% methanol (MeOH), 1% lactic acid, 16% hydrochloric acid (HCl), sterile distilled water (SDW), peramine hemisulfate (peramine), *n*-acetylloline.HCl (acetylloline), *n*-formylloline (d3) (formylloline), lolitrem B, janthitrem A, paxilline, terpendole E, terpendole C, ergovaline, ergotamine, ergocornine, ergocryptine, ergotaminine. Observations on days three and six (in rows) of the bioassay images are typical representatives of the five replicates.

Visual examination of agar well diffusion assays (Figure 1) suggest there is no effect on the growth of the three pathogens *Ceratobasidium* sp., *Drechslera* sp., and *Fusarium* sp., with no changes in growth area, mycelial density, or direction of growth, in the presence of any of the tested alkaloids. 

**Figure 2:**
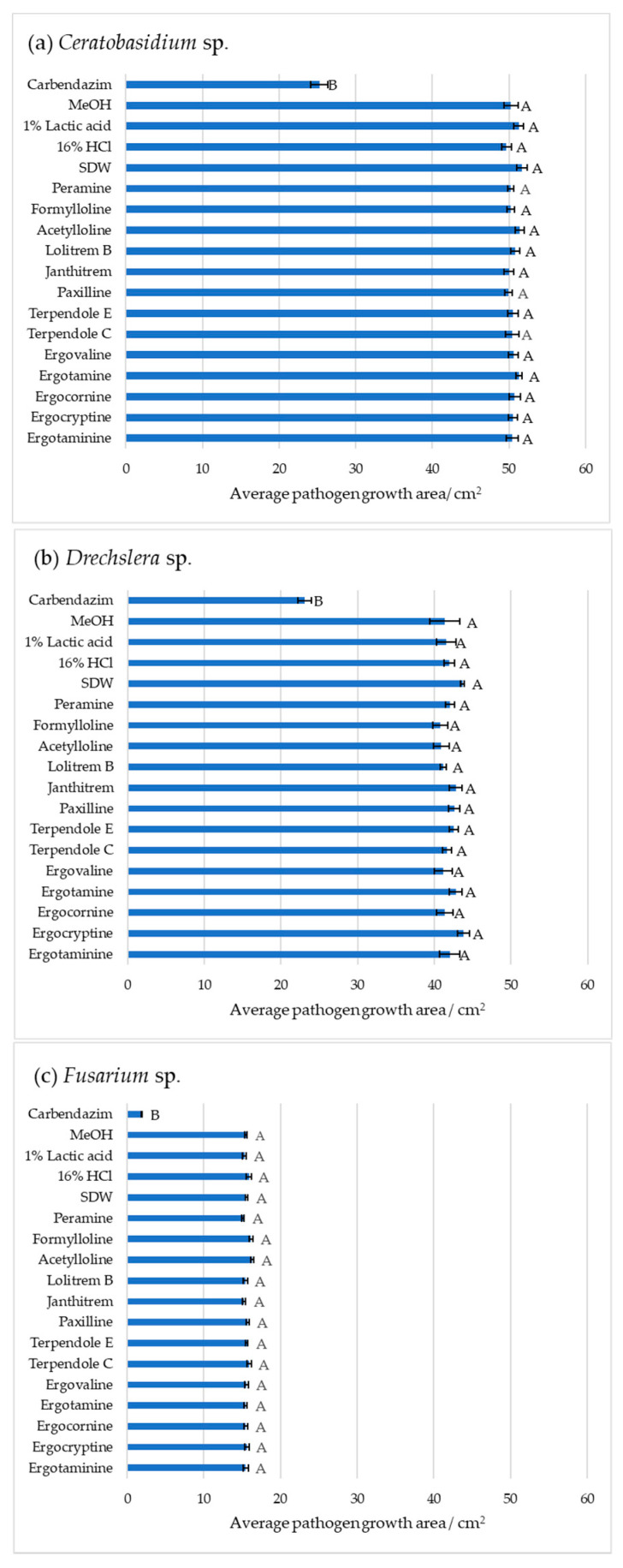
Pathogen growth inhibition by thirteen *Epichloë* sp. alkaloids and controls; carbendazim, 80% methanol (MeOH), 1% lactic acid, 16% hydrochloric acid (HCl), sterile distilled water (SDW), peramine hemisulfate (peramine), *n*-acetylloline.HCl (acetylloline), *n*-formylloline (d3) (formylloline), lolitrem B, janthitrem A, paxilline, terpendole E, terpendole C, ergovaline, ergotamine, ergocornine, ergocryptine, ergotaminine against (**a**) *Ceratobasidium* sp.; (**b**) *Drechslera* sp.; (**c**) *Fusarium* sp. Image analysis measured the growth area (cm^2^) of the pathogen in the agar well diffusion assay at day six. All data are mean ± standard error, *n* = 5. Significance was determined by one-way ANOVA and Tukey post-hoc test for pairwise comparison; *p* < 0.01 indicates significant inhibition. Means that do not share a letter are significantly different. In each instance Tukey post-hoc tests grouped the positive control, carbendazim, separately to all other treatment groups.

The average pathogen growth area obtained from image data analysis was used as a measurable indication of antifungal activity (Figure 2). One-way ANOVA of day six observations confirmed that the tested *Epichloë* sp. alkaloids did not significantly reduce pathogen growth compared to the negative controls. The following Tukey pairwise comparison *p* values, where *p* < 0.01 is significantly different, are for *Ceratobasidium* sp., *Drechslera* sp., and *Fusarium* sp., respectively, when compared to the corresponding negative control: peramine hemisulfate (*p* = 1, *p* = 1, *p* = 0.999), *n*-formylloline.d3 (*p* = 1, *p* = 1, *p* = 0.843), *n*-acetylloline.HCl (*p* = 0.939, *p* = 1, *p* = 0.997), lolitrem B (*p* = 1, *p* = 1, *p* = 1), janthitrem A (*p* = 1, *p* = 1, *p* = 1), paxilline (*p* = 1, *p* = 1, *p* = 1), terpendole E (*p* = 1, *p* = 1, *p* = 1), terpendole C (*p* = 1, *p* = 1, *p* = 0.997), ergovaline (*p* = 1, *p* = 1, *p* = 1), ergotamine (*p* = 1, *p* = 1, *p* = 1), ergocornine (*p* = 1, *p* = 1, *p* = 1), ergocryptine (*p* = 1, *p* = 0.932, *p* = 1), and ergotaminine (*p* = 1, *p* = 1, *p* = 1). Carbendazim (1000 ppm; parts per million), a systemic benzimidazole fungicide commonly used in laboratory assays and in this study as a positive control, strongly inhibited the growth of the three pathogens *Ceratobasidium* sp. (*p* < 0.001), *Drechslera* sp. (*p* < 0.001), and *Fusarium* sp. (*p* < 0.001) by significantly reducing growth area, (*p* < 0.01). Strong growth inhibition of *Ceratobasidium* sp. and *Fusarium* sp. was observed as early as the third day, while *Drechslera* sp. was moderately inhibited at day six. The negative controls of 80% methanol, 1% lactic acid, 16% hydrochloric acid, and sterile distilled water showed no signs of pathogen inhibition. These data confirmed that the *Epichloë* sp. alkaloids tested do not show antifungal activity against the three grass pathogens *Ceratobasidium* sp., *Drechslera* sp., and *Fusarium* sp.

## 3. Discussion

The antifungal activity of thirteen *Epichloë* sp. related alkaloids and intermediates from different compound classes (indole diterpenes, lolines, ergot alkaloids, and the pyrrolopyrazine alkaloid peramine) was assessed against three phytopathogenic fungi using in vitro agar well diffusion assays. No visible changes in pathogen growth were observed and image analysis confirmed there was no statistical difference between the growth areas of the pathogens in the presence of the pure compounds, namely peramine hemisulfate, *n*-formylloline-d3 (>98% pure), *n*-acetylloline hydrochloride (>98%), lolitrem B (>98%), janthitrem A (>95%), paxilline (>98%), terpendole E (>95%), terpendole C (>95%), ergovaline (≥97%), ergotamine (≥97%), ergocornine (>98%), ergocryptine (>98%), and ergotaminine (>98%), when compared to negative controls. This confirmed that these compounds do not possess antifungal activity at the concentrations tested against *Ceratobasidium* sp., *Drechslera* sp., or *Fusarium* sp. In contrast, the positive control Carbendazim, a fungicide commonly used in laboratory assays [34,35], strongly inhibited the growth of the three pathogens.

Visual observations from this study confirm previous findings by Siegel and Latch (1991) [18], and statistical analysis of quantified image data corroborates the findings. This study also further extends the observations made by Siegel and Latch (1991), to include *Epichloë* sp. (and related) alkaloids and pathway intermediates not previously investigated, such as ergovaline, ergocornine, ergotaminine, lolitrem B, janthitrem A, paxilline, terpendole E, and terpendole C.

The purity of the tested compounds, as described above, was greater than 95%. Each of the compounds used in this study have been shown to be in bioactive forms in other studies. For example, ergovaline, ergotamine, lolitrem B, paxilline, terpendole C, and terpendole E used in this study were previously used in investigations of mammalian toxicity in mice by Reddy et al. [6,36,37]. Paxilline and janthitrem A were used to investigate toxicity in mouse and insect models [32,38]. Ergot alkaloids ergotamine, ergocornine, ergocryptine, and ergotaminine have been widely used in investigations of mammalian toxicity [39]. Ergotamine, ergocornine, and ergocryptine are also known for their therapeutic application in medical conditions, e.g., migraine, uterotonics, cancer treatments, type II diabetes, and Parkinson’s disease [40]. In instances where deuterated or salt derivatives of the compounds were used, the compounds may not display the same properties as in their natural forms. However, in the case of peramine hemisulfate, *n*-formylloline-d3, and *n*-acetylloline hydrochloride, the salts dissociate in solution to their natural forms, which are known for their insecticidal activity [41,42,43]. The salts and solvents were also pre-tested for their effects on pathogen growth in agar well diffusion assays, and 80% methanol, 16% HCl, and 1% lactic acid were included in the bioassay as negative controls.

The concentrations of the test alkaloids used in this study also provide an accurate indication of the likely occurrence of bioprotection conferred by individual compounds under natural conditions. To make an informed choice for the concentrations tested, average in planta concentrations were obtained from glasshouse-maintained perennial ryegrass-endophyte symbiota using liquid chromatography-mass spectrometry (LCMS) based detection and analysis of *Epichloë* sp. endophyte alkaloids [44]. It is important to note that alkaloid concentration varies enormously under different field conditions, such as season, temperature, and local environment [45,46], as well as during host plant establishment and development [47]. There is also significant host plant genotype-endophyte strain interaction that impacts quantitative alkaloid concentrations [45,46]. In this study, we analyzed average alkaloid abundance in planta for selected perennial ryegrass–endophyte symbiota, i.e., NEA12 (epoxy janthitrem I), NEA23 (lolines), and SE (peramine, lolitrem B, paxilline, terpendole E, terpendole C and ergovaline), grown under controlled glasshouse conditions. This was to inform the concentrations used in the bioassay as the abundance of some of these alkaloids produced in the field, particularly indole diterpene pathway intermediates and lolines in perennial ryegrass, has not been published. However, alkaloid abundances of well characterized endophyte-derived alkaloids, such as lolitrem B, ergovaline, and peramine, are higher in the glasshouse-maintained plants measured in this study than what is generally observed in the field [48,49]. 

While perennial ryegrass infected with *Epichloë* sp. endophytes provides protection against insect pests and diseases and thus is beneficial to pastoral agricultural systems, some of the major alkaloids are also detrimental to grazing animals at concentrations that result in toxicity (i.e., lolitrem B, paxilline, terpendole C, epoxy-janthitrem I, ergovaline). These alkaloids would not be suitable as biocontrol agents at toxic concentrations [5,6]. Hence, while it is possible that at higher concentrations the alkaloids would display some antifungal activity, they would be neither safe nor biologically relevant. 

In a previous study by Fernando et al. (2020) [20], bioactive *Epichloë* sp. endophyte strains were identified in an in vitro screen for antifungal activity. A key observation was the differential bioactivity observed between three highly bioactive strains, namely NEA12, NEA23, and SE [20]. The three strains also exhibit different alkaloid profiles. NEA23 produces peramine, *n*-formylloline, and *n*-acetylloline, NEA12 produces epoxy janthitrems, and SE produces lolitrem B, peramine, and ergovaline [30,31,50]. This study establishes that the known, and well characterised in terms of animal and insect toxicity, endophyte-derived alkaloids are not responsible for the antifungal properties previously observed. It is now important to identify and characterise the compounds responsible for the antifungal activity of novel strains.

## 4. Conclusions

*Epichloë* sp. endophytes provide pasture and turf grasses with resistance to insect pests, animal herbivory, and disease resistance. For pastoral agriculture, the endophytes should maintain their insect and disease protection but contain little or none of the animal toxins that can cause disease in grazing animals. Although the compounds responsible for animal toxicity are largely known, the compounds responsible for disease resistance are not yet thoroughly described. In this study, the major alkaloids were tested for their in vitro antifungal activity. None of the tested compounds exhibited in vitro bioactivity towards the three phytopathogens tested. Since known alkaloid metabolites are not responsible for the antifungal activity expressed by *Epichloë* sp. endophyte strains, the bioactive metabolites are yet to be discovered. While this study provides an opportunity to rule out the contribution of alkaloids known to be produced by *Epichloë* endophyte strains, it opens avenues to explore novel metabolites produced by *Epichloë* endophytes. 

## 5. Materials and Methods

### 5.1. Plant Material

All plant material was obtained from a glasshouse maintained (natural day lengths and a mean temperature of 22 °C) collection at Agriculture Victoria Research, Bundoora, Victoria, Australia.

### 5.2. Plant Pathogens

All pathogens (Table 1) were obtained from the National Collection of Fungi, Bundoora Herbarium, Victoria. Pathogens were stored as solid cultures on potato dextrose agar (PDA) plates (90 mm × 14 mm) (Sigma-Aldrich, Castle Hill, NSW, Australia) at 22 °C in the dark, and sub-cultured every two months to maintain stocks.

The genus of the pathogens used in this study was confirmed by internal transcribed spaces (ITS) sequence analysis described by Fernando et al. (2020) [20]. 

### 5.3. Chemicals

Compounds were obtained from either commercial suppliers or isolated in-house as described in Table 2. In instances where an identical *Epichloë* related compound was not available, the deuterated, or salt derivative of the compound was examined. Stock solutions of compounds peramine hemisulfate, deuterated *n*-formylloline (*n*-formylloline-d3), *n*-acetylloline hydrochloride, janthitrem A, paxilline, terpendole E, terpendole C, ergocornine, ergocryptine, ergotaminine in 4:1 (*v*/*v*) methanol: water (H_2_O) (Fisher chemicals, Fair Lawn, NJ, USA) following manufacturer’s instruction. Ergovaline isolated from perennial ryegrass seeds by Reddy et al. [6] and ergotamine were pre-stored in a stock solution of 1% lactic acid (neat lactic acid, Sigma Aldrich, St. Louis, MO, USA, diluted with ultrapure distilled water, Invitrogen, Waltham, MA, USA). Lolitrem B isolated from perennial ryegrass seeds by Reddy et al. [56] was in 4:1 (*v*/*v*) methanol: H_2_O stock solution. All compounds were diluted to the desired concentrations (Table 2) in stock solution. For the positive control, a 1000 ppm solution of antifungal compound carbendazim (methyl benzimidazol-2-ylcarbamate; 97% pure; Sigma-Aldrich, Castle Hill, NSW, Australia), a systemic benzimidazole fungicide commonly used in both laboratory studies as well as in the field to control a broad range of diseases in crops [34,35,57,58], was prepared in 4:1 (*v*/*v*) methanol: H_2_O, 1% lactic acid (neat lactic acid, Sigma Aldrich, diluted with ultrapure distilled water) and 16% hydrochloric acid (HCl) (37% HCl, Scharlau Chemie S.A., Spain, diluted with ultrapure distilled water) was used [59]. 

### 5.4. Agar Well Diffusion Assay

Bioassays were conducted on PDA plates (90 mm × 14 mm). A 4 mm cork borer was used to make two 4 mm diameter wells 2 cm apart. The bottoms of the wells were then sealed using 25 µL of PDA media to ensure the compound was retained closer to the surface. A 5 × 5 mm plug of pathogenic fungal mycelia was transferred onto the centre of the PDA media plate and each agar well was filled with 20 µL of the compound solution. The bioassay plates were prepared in replicates (*n* = 5). Plates were incubated at 22 °C in the dark for eight days and observations were taken daily from day three. Two negative controls and one positive control were prepared. For the negative controls, the agar wells were filled with either 20 µL of sterile distilled water or 4:1, *v*/*v* methanol: H_2_O or 1% lactic acid or 16% HCl. Positive controls were prepared by filling the agar wells with 20 µL of carbendazim [20]. Pathogen growth area (cm^2^) was analysed using ImageJ 1× software (NIH, Bethesda, MA, USA). Image data of the sixth day of observations were used for the measurement of pathogen growth. One-way ANOVA was performed using Minitab^®^ 19 Statistical Software (Minitab, LLC, State College, PA, USA). Tukey post hoc comparison tables at 99% confidence level were used to determine significant differences in antifungal activity.

## Figures and Tables

**Table 1:** Summary of pathogens selected for this study.

Species	Host/Source	Accession Number	Disease	Effect on Pasture/ Seed Production	References
*Ceratobasidium* sp.	*Triticum aestivu*m (wheat)	VPRI 22537	yellow patch/ sharp eyespot	Reduced yield	[51,52,53]
*Drechslera* sp.	*Briza maxima* (rattle grass)	VPRI 12962	brown blight and net blotch	Dry matter and herbage yield reduction	[10,54]
*Fusarium* sp.	*Lolium perenne* (perennial ryegrass)	VPRI 43403	*Fusarium* patch	Reduced yield and dry matter	[55]

**Table 2:** Purified compounds and concentrations used in bioassay.

Biosynthesis Pathway	Purified Compound (Purity, %)	Chemical Formula	Concentration Used in Bioassay (ppm)	Concentration in Planta ^1,3^ (ppm)	Source
Peramine	Peramine hemisulfate	C_12_H_17_N_5_O.H_2_SO_4_	100	52.24 ± 3.10	Toronto Research Chemicals, North York, ON, Canada
Loline	*n*-Formylloline-d3 (>98%)	C_9_H_11_D_3_N_2_O_2_	500	427.92 ± 26.62	Toronto Research Chemicals, North York, ON, Canada
	*n*-Acetylloline. Hydrochloride (>98%)	C_10_H_16_N_2_O_2_.HCl	250	typically trace levels in perennial ryegrass ^2^	Toronto Research Chemicals, North York, ON, Canada
Indole diterpene	Lolitrem B (>98%)	C_42_H_55_NO_7_	10	4.88 ± 0.64	(Reddy et al., 2019) [56]
	Janthitrem A (>95%)	C_37_H_47_NO_6_	50	11.86 ± 0.85	BioAustralis, Smithfield NSW, Australia
	Paxilline (>98%)	C_27_H_33_NO_4_	10	0.24 ± 0.05	Sigma-Aldrich, St. Louis, MO, USA
	Terpendole E (>95%)	C_28_H_39_NO_3_	10	0.061 ± 0.01	Sigma-Aldrich, St. Louis, MO, USA
	Terpendole C (>95%)	C_32_H_41_NO_5_	10	1.33 ± 0.23	Sigma-Aldrich, St. Louis, MO, USA
Ergot alkaloid	Ergovaline (≥97%)	C_29_H_35_N_5_O_5_	10	0.40 ± 0.08	(Reddy et al., 2020) [6]
	Ergotamine (≥97%)	C_33_H_35_N_5_O_5_	10	NA	LGC Standards, Wesel, Germany
	Ergocornine (>98%)	C_31_H_39_N_5_O_5_	10	NA	LGC Standards, Wesel, Germany
	Ergocryptine (>98%)	C_32_H_41_N_5_O_5_	10	NA	LGC Standards, Wesel, Germany
	Ergotaminine (>98%)	C_33_H_35_N_5_O_5_	10	NA	LGC Standards, Wesel, Germany

^1^ Average alkaloid concentrations ± SEM in planta are from leaf material harvested from glasshouse maintained perennial ryegrass-endophyte symbiota. Selected endophytes NEA12 (*Lp*TG-3; epoxy janthitrem I, *n* = 114), NEA23 (*Fa*TG-3; lolines, *n* = 52), and Standard Endophyte (SE; *Epichloë festucae* var. *lolii*; peramine, lolitrem B, paxilline, terpendole E, terpendole C and ergovaline, *n* = 40); NA, not applicable to *Epichloë* sp., these compounds are produced by *Claviceps purpurea*. ^2^ Average concentrations of *n*-acetylloline in tall fescue-NEA23 symbiota are 119.9 ± 13.24 (*n* = 23). Concentrations are indicated as parts per million (ppm). ^3^ Alkaloid concentrations observed in glasshouse-maintained plants in this study are higher than the maximum concentrations generally observed under field conditions.

## Data Availability

The data presented in this study are contained in the article.
